# False Impressions

**Published:** 1889-01-12

**Authors:** Caroline Fothergill

**Affiliations:** Author of "An Enthusiast," "A Voice in the Wilderness," etc.


*(To be continued.)*

### FALSE IMPRESSIONS.

By Caroline Fothergill, Author of "An Enthusiast," "A Voice in the Wilderness," etc.

*(Continued from p. 224.)*

### Chapter XIV.—Telling the Truth.

Beatrice was glad when she was alone in her cab. She felt very much shaken by all that had taken place, and when she had withdrawn her hand from George's arm and walked alone, the effort she had made had been far greater than he had had any idea of. Now it was an inexpressible relief to her to be able to sit down and feel that she need no longer keep up appearances. As she became more herself, she began to think, and her thoughts were not tranquillising. Although she tried hard, she could not recollect all that had passed, and the very vagueness of her recollections increased their significance in her eyes. She recalled the expression in George's face, the tone of suppressed passion in his voice as he spoke to her, and which she either did not or would not believe to be sincere. There was her own wish to lean upon him, her betrayal of that wish, her admission that she was glad he was there. She was very proud, and the feeling that when not wholly mistress of herself, she had yielded to the influence which she was constantly fighting against galled her inexpressibly. What would he think of it, how interpret the sudden change in her manner, and how much more which she could not remember had taken place? She felt that things which she ought not to have allowed had taken place; she condemned herself for having permitted their intercourse to overstep the bounds which had hitherto been set to it, and which she had persuaded herself were both right and necessary. She had a very strong feeling as to the relations which should exist between two people situated as she and George Murray were situated; and not even Laura had ventured to suggest that constantly meeting and working together, as they were, certain consequences were probable, if not inevitable. She had decided in her own mind that they could be friends only, and friends only they should remain. How, she could not say, but she had an uneasy feeling that friendship alone had not been the motive for what George had done. Still, it had been for her to suppress any signs of a more intimate feeling, and she had not suppressed them. The chief fault lay with her, and she was very angry with herself.

She reached home just as Laura, who had been paying a round of calls, got out of the carriage, and Mrs. Ledward greeted her at once.

"How hot it is!" she said. "I am so glad you have taken a cab. It is not fit to walk. I was just wondering whether I dare risk sending the carriage to meet you on the usual road."

Beatrice paid and dismissed the cabman, and then answered:

"I was tired; I could not have walked home."

She spoke abruptly, as if she did not wish the subject to be continued; but Laura, nothing dismayed, went on.

"You look tired," she said, surveying her friend's face critically. "You're as white as a sheet; somehow, you give me the impression of having passed through a crisis. You have been through a crisis," she added, her keen eyes taking in every detail of Beatrice's appearance. "Look at your dress! Where have you been? What have you been doing?"

Beatrice had completely forgotten her dress, which was dirty and crushed. "Utterly ruined," Laura said.

"I will tell you after dinner," she said, a little impatiently. "There is not time now, and I am tired."

So, after dinner she gave a sketch of her adventure: she did not intend to go into any details, but Laura's penetrating questions soon extracted the whole truth from her. She did not mention George's name. Laura heard her with attention and respect; but the whole story moved her more to blame than sympathy.

"I wish you would not do such things," she said, crossly, at the end of the history. "You never seem to know where to draw the line. Some day you will be finding yourself in the middle of a regular street row, and perhaps summoned to appear in Court."

"Then I suppose you would have left the dog to its fate?" said Miss Stanford.

"Yes," said Mrs. Ledward, hardily, for she gauged Beatrice's feelings by the tone in which she asked the question. "I should have felt sorry for it, but I should not have interfered. I should have got it out of my sight and my mind as soon as I could. There was the boy it belonged to to look after it, and no doubt a policeman somewhere about. I should not have interfered."

Beatrice said nothing, but the look in her face expressed the reverse of approval, and Laura went on—

"I am thankful no one we knew was about, and that it did not happen in our part of the town. You will get a name for being eccentric if you will do these things."

"As it happened, there was someone I knew about," said Beatrice, with some constraint—for she was most unwilling to mention George's name, and yet she would not leave Laura under a false impression—"Mr. Murray was there."

If Laura had been a man she would have whistled. As it was, she preserved as stolid a countenance as was possible with features like hers, and merely said,

"Indeed!"

"He walked with me as far as a cab-stand, and put me into a cab."

"He could not have done less," was Laura's calm reply.

The next afternoon the two ladies were seated in the drawing-room. The heat of the previous day had culminated late in the evening in a thunderstorm. It had rained all night and all this present day. No callers had been, and it was getting too late to expect any. Laura was yawning, and saying how dull it was, when suddenly the door opened, and Mr. Murray and Mrs. Lester were announced.

So great was Laura's astonishment that she arrested her yawn midway, and sat with her lips apart, her little plump, white hand held before them, and her bright, dark eyes opened, and almost fixed in their expression. Still, she remained alive to what was passing, She saw Mrs. Lester's almost aggressively stiff bearing, and at once decided that she was making the call under compulsion. She took in the subdued eagerness of George's manner; and she also saw with a feeling of dismay and wonderment that Beatrice, in the twinkling of an eye, had put on a manner as frigid as it could be consistent with politeness, and was speaking in a tone the exceptional clearness and precision of which Laura knew well.

"What is the meaning of this?" she asked herself. "If they had come to insult her, she could not look more forbidding. I believe she thinks they have come to insult her, and indeed, why have they come? I suppose this is a result of yesterday."

It was a strange call. Laura often thought of it in after days. She at once took upon herself the burden of entertaining Mrs. Lester, and, had she been left to herself, would have carried even that task triumphantly through. She intended to give George full opportunity to talk to Beatrice, and she saw at a glance that he was perfectly willing to take advantage of the opportunity. But Beatrice kept interfering with this plan. Again and again she ignored Laura's endeavours to divide the party, and insisted upon joining in the conversation between Mrs. Ledward and Mrs. Lester. She spoke no more to George than politeness demanded, and his efforts to draw her into conversation were entirely unsuccessful. Beatrice was seconded by Mrs. Lester, and Laura was soon highly amused to find that she and George were apparently engrossed in a *tête-à-tête*, while Beatrice and Mrs. Lester carried on a halting and laboured dialogue. She could not hear what they were saying, but she could see that Mrs. Lester spoke more than her hostess.

They did not stay long; and as soon as they had gone, Laura leaned back in her chair and went into a fit of laughter.

"People of weight, decidedly!" she said. "What on earth brought them here?"

"I am utterly at a loss to imagine," said Beatrice.

"Well, I am going to hazard a guess," said Laura, courageously. "I have a very clear notion as to what brought them—or at least him—here."

"Indeed," was all Beatrice replied.

"Yes," continued Laura, undauntedly: "It was you who brought him."

Beatrice was silent, and neither spoke for a long time, then suddenly Laura began again in rather a different tone.

"You don't often hear the truth, Beatrice; rich people seldom do, it is one of the penalties they have to pay for being rich. But I'm going to tell it you now; Mr. Murray is in love with you."

Beatrice blushed crimson, and for a moment could not speak. Then she recovered herself, and said in a voice which was not quite steady—

"You do not know what you are talking about, and I beg you will not say such a thing again."

"I shall say it again, because it is true, and sooner or later you will acknowledge it. Why do you suppose he called this afternoon?"

Beatrice, with the recollection of the previous afternoon vividly before her, felt as if she were being brought to bay, and answered—

"Probably to see what I looked like after yesterday. He thought me very unwomanly for doing what I did."

"He came because he could not stop away. He wanted to see you again, to ask how you were, to speak to you and hear you speak—to be in the same room with you."

Beatrice's colour had varied two or three times while Laura was speaking. When she had finished, she said—

"You are quite mistaken: he has no regard for me at all; and if he had, whether he had or had not, he had no right to come this afternoon without invitation or encouragement. I am not here for any man to call upon me who likes; his coming was an unpardonable impertinence."

Laura sat aghast at this sudden outburst of feeling. Beatrice's cheeks were flushed; tears sparkled in her eyes. She looked very beautiful. Laura was so struck with her appearance that she forgot to speak, and meanwhile Beatrice went on—

"He has often enough given me to understand that he has not a high opinion of me, but I did not think he would take such a liberty as to force himself into my house. He must think even more poorly of me than I believed. My eyes are opened."

"How you harp upon his opinion," said Laura impatiently. "What is his opinion to you?"

"He is an honest man, I suppose; and therefore I value his opinion."

"What nonsense, what arrant nonsense, you are talking—with your opinion, and your respect, and your honest men! If any other woman was to talk such preposterous nonsense, I should say she deserved to be punished. Who cares for an honest man? He is the man who loves you, and what is more, the man you would love if you would only let yourself; only you treat him as if you wished him a thousand miles away.'

"Laura!" came from Beatrice in a warning voice, but she made no other reply.

"Oh, you need not warn me," retorted Mrs. Ledward, with a little excitement. "I know that I am treading on volcanoes, and precipices, and hidden pitfalls, and snares and delusions—but it is true all the same; and if you were honest, you could not deny it."

"I do deny it, emphatically."

"Why 'emphatically?' Why speak in that defiant tone? If I am wrong, it is enough to say so. A simple 'no' is enough to oppose an untruth with"

"I think," said Beatrice rather grandly, "that you are taking a liberty in speaking about Mr. Murray at all."

"Of course!" said Laura, sarcastically. "People always take liberties when they speak the truth. 'Reason is never inconvenient but when it comes to be applied.' We all know that."

There was another silence, and then Beatrice said rather hesitatingly—

"I think I shall go away from home."

"Do," taunted Laura. "Rush! fly! It is the best thing you can do. There is time yet to catch the night express to London; we could be anywhere to-morrow. When Mr. Murray hears that you have run away from him, his opinion of you will be immensely higher."

"There is no need to pursue the subject," said Beatrice, with dignity. "We take opposite views of the question, and you don't seem able to speak about it without losing your temper and becoming abusive. I repeat, you are quite mistaken about both my feelings and Mr. Murray's."

"My dear," said Laura, who felt both angry and disappointed, "you have got another bone in your throat."

